# Down-regulation of circular RNA ITCH and circHIPK3 in gastric cancer tissues

**DOI:** 10.3906/sag-1806-50

**Published:** 2018-04-18

**Authors:** Sara GHASEMI, Modjtaba EMADI-BAYGI, Parvaneh NIKPOUR

**Affiliations:** 1 Department of Genetics and Molecular Biology, Faculty of Medicine, Isfahan University of Medical Sciences, Isfahan Iran; 2 Research Institute of Biotechnology, Shahrekord University, Shahrekord Iran; 3 Department of Genetics, Faculty of Basic Sciences, Shahrekord University, Shahrekord Iran; 4 Child Growth and Development Research Center, Research Institute for Primordial Prevention of Non-Communicable Disease, Isfahan University of Medical Sciences, Isfahan Iran

**Keywords:** Gastric cancer, circular RNAs, *cir_ITCH*, *circHIPK3*, gene expression

## Abstract

**Background/aim:**

Gastric cancer (GC) is one of the major causes of cancer mortality worldwide. As a novel type of endogenous noncoding RNAs, circular RNAs (circRNAs) are formed by a covalent link between 5’ and 3’ ends. They are very stable and abundant in eukaryotes. As there were no reported studies on the expression profiles of *circular RNA ITCH (cir-ITCH) *and *circHIPK3*in GC, in the current study, we aimed to delineate the expression profiles and clinicopathological relevance of these two circRNAs in GC tissues compared to their paired adjacent noncancerous tissues.

**Materials and methods:**

Quantitative real-time polymerase chain reaction was performed to evaluate *cir_ITCH* and *circHIPK3* expression in 30 paired gastric cancer tissues. The clinicopathological relevance of these two circular RNAs’ expression levels with gastric cancer was further examined**.**

**Results:**

Our results showed that the expression of *cir_ITCH *and *circHIPK3* were significantly downregulated in GC tumoral tissues compared with their paired adjacent nonneoplastic counterparts. Further analyses showed that *cir_ITCH *and *circHIPK3* expression levels were related with numerous clinicopathological features****of tumoral tissues.

**Conclusion:**

*Cir_ITCH* and *circHIPK3* may have imperative roles in GC and serve in the future as potential prognostic biomarkers in GC.

## 1. Introduction

Gastric cancer (GC) is one of the unresolved causes of cancer mortality worldwide with a high rate of incidence and death (1,2). It is the second and fourth most common cancer leading to a high death rate in men and women, respectively (3,4). Lack of reliable diagnostic methods in early stages of GC denies the majority of patients an effective treatment (3,5). Accordingly, to overcome these problems, it is critical to find novel biomarkers to improve the chance to diagnose GC in its early stages.

As a novel type of endogenous noncoding RNAs, circular RNAs (circRNAs) are formed by a covalent link between 5’ and 3’ ends (6–8). They are very stable and abundant in eukaryotes (9–11). Recent reports show that circRNAs can function as microRNA sponges, regulate gene expression, linear RNA transcription and protein production (7). Association between circRNAs and several diseases like atherosclerotic vascular disease (12), Alzheimer (13), and various cancer types (14) has been documented and could represent these molecules as attractive novel biomarkers.

*cir-ITCH* is a circular RNA which is derived from Itchy E3 ubiquitin protein ligase (*ITCH*) gene (10,15). Deregulation of *cir-ITCH* has been recently reported in esophageal squamous cell carcinoma (ESCC) (16), colorectal cancer (17), and lung cancer. Further functional studies showed that *cir-ITCH* can act as microRNA sponge thus increasing the level of parental gene, *ITCH* (15). 

In 2016, by characterizing circRNA, transcripts using RNA-sequencing (RNA-seq) from six normal tissues (brain, colon, heart, liver, lung, and stomach) and seven cancerous tissues including gastric cancer, Zheng et al. introduced an abundant circRNA derived from Exon2 of the *HIPK3* gene, termed* circHIPK3*. Their functional assays revealed that *circHIPK3* may function to modulate the growth of human cells (18). 

As there were no reported studies on the expression profiles of these two circular RNAs in gastric cancer, in the current study, we aimed to delineate the expression profiles of *cir-ITCH* and *circHIPK3* in gastric cancer tissues compared to their paired adjacent noncancerous tissues. Then, the clinicopathological relevance of these two circular RNAs with gastric cancer was further examined.

## 2. Materials and methods

### 2.1. Clinical specimens

A total of 30 pairs of gastric cancer and matched adjacent nontumoral tissues were obtained from patients with gastric cancer. The specimens were collected by the Iran National Tumor Bank, which is funded by the Cancer Institute of Tehran University, for Cancer Research (19–21). There, the tissues are immediately snap-frozen in the liquid nitrogen. Informed written consent was taken from the patients by Iran National Tumor Bank. The study protocol was approved by the Ethics Committee of Isfahan University of Medical Sciences and was in accordance with the Helsinki Declaration. The clinical staging of the tumor samples was based on the seventh edition of the American Joint Committee on Cancer classification (AJCC) cancer staging manual for stomach (22).

### 2.2. Total RNA extraction and complementary DNA (cDNA) synthesis

TRIzol® reagent (Invitrogen, California, USA) was used to extract total RNA from powdered gastric cancer tissues, following the manufacturer’s instructions. One percent agarose gel electrophoresis was used to assess the quality of the RNA. Purity and quantity of the total RNA were determined with Nanodrop instrument (Nanolytik, Düsseldorf, Germany). DNase treatment was performed by using DNase set (Fermentas, Vilnius, Lithuania) for eliminating genomic DNA. cDNA was synthesized by using PrimeScriptTM RT reagent Kit (TaKaRa, Kusatsu, Shiga, Japan) according to manufacturer’s protocol.

### 2.3. Quantitative real-time PCR and DNA sequencing

Applying the relative quantitative real-time RT-PCR, the expression levels of *cir_ITCH* and *circHIPK3* were assessed compared to *GUSB* (β-Glucuronidase) as an internal control (23). Divergent primers, rather than convergent primers, were designed with GeneRunner software, version 4.0 to amplify the circular RNAs. Basic local alignment search tool (BLAST) (http://blast.ncbi.nlm.nih.gov/Blast.cgi) was used for confirmation of unity attachment of divergent primers to genome. A set of convergent primers in an opposite direction were designed to amplify only the linear forms. The sequences of primers are listed in Table 1. RealQ Plus 2x Master Mix, green (high ROX) (AMPLIQON, Odense M, Denmark) was applied in an Applied Biosystems StepOnePlus™ instrument for PCR amplification. The amplification conditions consisted of an initial denaturation at 95 °C for 2 min, then 30 cycles of denaturation at 95 °C for 30 s, annealing at 55.9 °C for *cir_ITCH* and *circHIPK3* and at 60 °C for *GUSB *genes for 60 s, and the extension step for 30 s at 72 °C. Furthermore, the PCR products of some samples were sequenced with an Applied Biosystems 3730XL sequencer (Macrogen, Seoul, South Korea) to verify the specific amplification of circular RNAs.

**Table 1:** The sequences of primers used in the current study.

Characteristics	Numbers(#30)(%)	circHIPK3(mean ± SEM*)	P-value
Gender Male Female	17(56.67)13(44.33)	0.29 ± 0.660.37 ± 0.46	0.25
Age (years) ≥70 <70	15(50.00)15(50.00)	1.93 ± 0.44−1.28 ± 0.55	0.002**
Depth of invasion T2 T3-T4	1(3.33.00)29(96.67)	−3.21 ± 0.45 ± 0.57	0.14
N classification NX-N0 N1 N2-N3	6(20.00)11(36.67)13(43.33)	−1.01 ± 0.811.52 ± 0.44−0.07 ± 0.53	0.16
M classification MX M0 M1	5(16.67)19(63.33)6(20.00)	−0.47 ± 0.231.31 ± 0.54−1.89 ± 0.66	0.045**(M0 vs. M1)
TNM stage I-II III IV	16(53.33)8(26.67)6(20.00)	0.86 ± 0.560.92 ± 0.44−1.89 ± 0.66	0.16
Perineural invasion Negative Positive	12(40.00)18(60.00)	0.08 ± 0.660.57 ± 0.51	0.28
Lymphatic invasion Negative Positive	6(20.00)24(80.00)	0.91 ± 0.530.24 ± 0.46	0.20
Tumor size (cm) ≥5 <5	25(83.33)5(16.67)	0.23 ± 0.570.79 ± 0.64	0.40
Tumor grades I II III	9(30.00)8(26.67)13(43.33)	−0.84 ± 0.670.98 ± 0.610.73 ± 0.48	0.25
Tumor types Diffuse Intestinal	14(46.67)16(53.33)	0.78 ± 0.49−0.07 ± 0.64	0.38

### 2.4. Statistical gene expression analysis

The ΔCt method was applied to relatively quantify the levels of gene expressions. All experiments were performed at least three times and expressed as means ± standard error of mean (SEM). To check the normal distribution of samples, Kolmogorov–Smirnov test was applied. Student’s t-test, analysis of variance (ANOVA), and chi-square tests were performed to examine statistical significances. Data were analyzed by SPSS software, version 16.0 (SPSS, Chicago, IL, USA) and P-values less than 0.05 were considered statistically significant.

## 3. Results

### 3.1. PCR optimization of the cir_ITCH and circHIPK3

**Figure 1:**
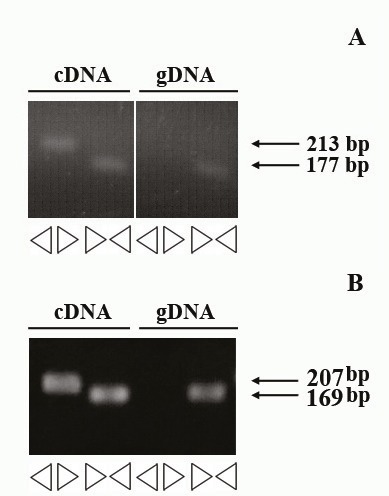
Identification of circHIPK3 and cir_ITCH in a human lung adenocarcinoma epithelial cell line (A540). A) RT-PCR products with divergent and convergent primers of circHIPK3 verified that specificity of designed primers. B) Specific divergent primers of cir_ITCH could amplify a 207 bp PCR product using a cDNA as a PCR template but not a genomic DNA (gDNA).

We designed two sets of primer pairs: a divergent primer set for only amplification of the circular form and a convergent one to amplify the linear form. We utilized the cDNA and DNA of a human lung adenocarcinoma epithelial cell line (A540) for PCR optimization. As expected, no amplification was seen with divergent primers on DNA template, whereas we could detect the expected bands corresponding to circular forms of RNAs (Figure 1). Products giving a band were sequenced by conventional Sanger capillary methods and compared to the reference sequence. Sanger sequencing of the RT-PCR products of *cir_ITCH* and *circHIPK3 *showed that convergent primers could specifically amplify the circular forms of RNAs (data not shown).

### 3.2. cir_ITCH and circHIPK3 expression levels were significantly downregulated in GC tumoral tissues and were significantly correlated with various clinicopathological parameters

**Figure 2:**
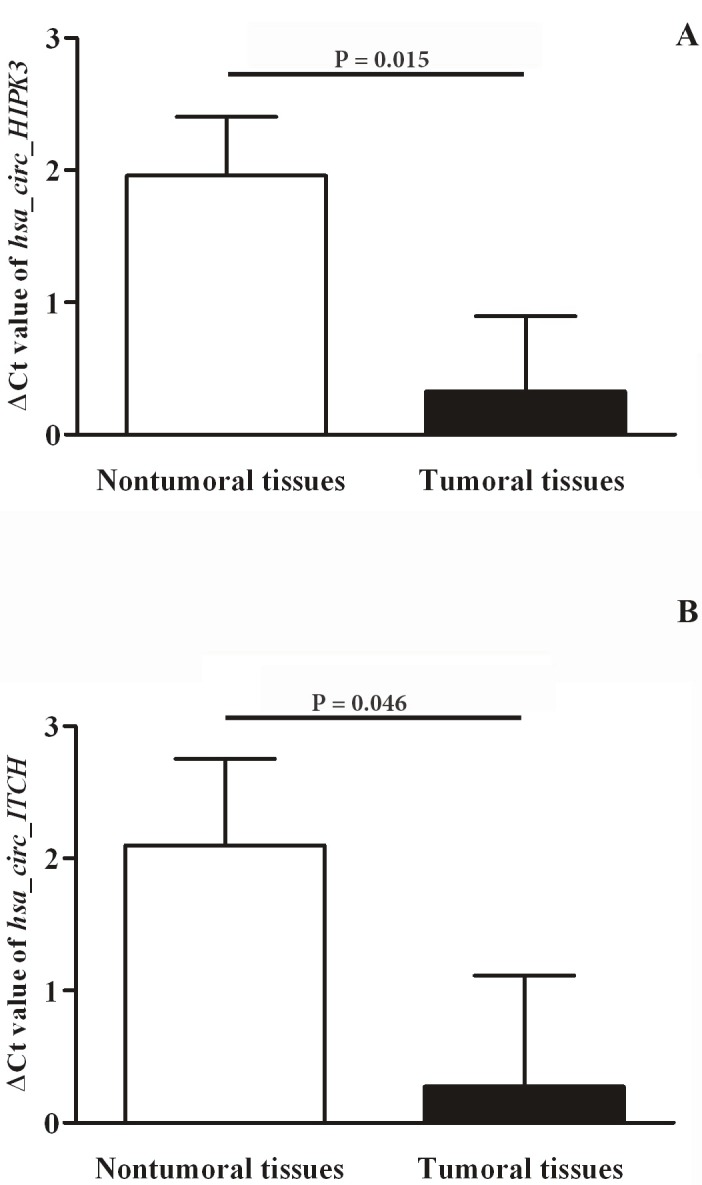
Relative expression of circHIPK3 and cir_ITCH in tumoral and nontumoral gastric tissue samples. A higher ΔCt value shows higher relative expression levels. Error bars stand for standard error of mean (SEM). A) circHIPK3, B) cir_ITCH.

The results of real-time qRT-PCR experiments showed that expression of both *cir_ITCH* and *circHIPK3* were significantly downregulated (P = 0.015 and P**= 0.046, respectively) in GC tumoral tissues compared with their paired adjacent nontumoral tissues (Figure 2). Analyzing melting curves showed single peaks with neither primer dimers nor nonspecific amplification, further confirming the specificity of *cir_ITCH *and *circHIPK3* amplifications.

Further analyses showed that *circHIPK3* expression was related with numerous clinicopathological features****of tumoral tissues. As shown in Table 2, *circHIPK3* expression level was significantly associated with age (P = 0.002) and M classification (P = 0.045). GC patients were further classified into two groups based on the median value of *circHIPK3* expression in tumoral tissues. *circHIPK3* expression levels stratified based on median value showed a significant association with age (P = 0.01) (Table 3). Same analyses on *cir_ITCH* showed a significant correlation between circRNA expression levels and age (P = 0.0005) and tumor grades (P = 0.02) (Tables 4 and 5). We furthermore tested if there is any correlation between gene expression levels and the anatomical location of tumors within the stomach. Although the stomach is anatomically divided into four regions, i.e. the cardia, fundus, body, and pylorus, the two main regions in our patient cohort were the cardia and the main body. Therefore, we compared the gene expressions between these two groups (body vs. cardia) and found no statistically significant difference in gene expressions of the two examined circular RNAs between tumors in the main body of the stomach and the ones in cardia (1.06 ± 1.03 vs. −1.54 ± 1.67 for *cir_ITCH* (P**= 0.20) and 1.14 ± 0.62 vs. −0.57 ± 1.18 for *circHIPK3* (P**= 0.22)).

**Table 2:** The relationship between circHIPK3 expression level (based on mean ± SEM gene expression level) in GC tissues with clinicopathological parameters.

Characteristic	Number (#30)	circHIPK3 expression		P-value
		Low (#15)	High (#15)	
Gender Male Female	1713	96	87	0.50
Age (years) ≥70 <70	1515	411	114	0.01**
Depth of invasion T2 T3-T4	129	114	-15	0.50
N classification NX-N0 N1 N2-N3	61113	348	375	0.23
M classification MX M0 M1	5196	474	1122	0.09(M0 vs. M1)
TNM stage I-II III IV	1686	744	942	0.31
Perineural invasion Negative Positive	1218	69	69	0.50
Lymphatic invasion Negative Positive	624	213	411	0.32
Tumor size (cm) ≥5 <5	255	123	132	0.50
Tumor grades I II III	9813	645	348	0.21
Tumor types Diffuse Intestinal	1416	69	87	0.35

* A higher ΔCt value indicates higher expression. ** Statistically significant.

**Table 3:** The relationship between circHIPK3 expression level (as divided into two groups based on the median of ΔCt) in GC tissues with clinicopathological parameters

Characteristic	Number (#30)(%)	cir_ITCH expression		P-value
		Low (#15)	High (#15)	
Gender Male Female	1614	78	96	0.36
Age (years) ≥70 <70	1416	213	123	0.0005**
Depth of invasion T2 T3-T4	129	114	015	0.50
N classification NX-N0 N1 N2-N3	61113	555	168	0.08
M classification MX M0 M1	5196	573	0123	0.28(M0 vs. M1)
TNM stage I-II III IV	1686	744	942	0.31
Perineural invasion Negative Positive	1218	510	78	0.35
Lymphatic invasion Negative Positive	624	213	411	0.34
Tumor size (cm) ≥5 <5	255	123	132	0.50
Tumor grades I II III	9813	744	249	0.04**
Tumor types Diffuse Intestinal	1416	510	96	0.13

* A higher ΔCt value indicates higher expression. ** Statistically significant.

**Table 4:** The relationship between cir_ITCH expression level (based on mean ± SEM gene expression level) in GC tissues with clinicopathological parameters

Characteristics	Numbers (#30)(%)	cir_ITCH (mean±SEM*)	P-value
Gender Male Female	16(53.33)14(46.66)	0.31 ± 0.930.23 ± 0.75	0.42
Age (years) ≥70 <70	14(46.66)16(53.33)	2.66 ± 0.76−2.11 ± 0.69	0.0005**
Depth of invasion T2 T3-T4	1(3.33)29(96.67)	0.45 ± 0.27 ± 0.85	0.50
N classification NX-N0 N1 N2-N3	6(20.00)11(36.67)13(43.33)	−1.18 ± 1.250.89 ± 0.870.42 ± 0.61	0.45
M classification MX M0 M1	5(16.67)19(63.33)6(20.00)	−2.24 ± 0.331.76 ± 0.63−1.62 ± 1.34	0.16(M0 vs. M1)
TNM stage I-II III IV	16(53.33)8(26.67)6(20.00)	1.19 ± 0.680.41 ± 0.60−2.35 ± 1.35	0.23
Perineural invasion Negative Positive	12(40.00)18(60.00)	−0.34 ± 0.960.68 ± 0.77	0.44
Lymphatic invasion Negative Positive	6(20.00)24(80.00)	3.28 ± 1.60−0.04 ± 0.89	0.08
Tumor size (cm) ≥5 <5	25(83.33)5(16.67)	−0.02 ± 0.891.72 ± 0.49	0.19
Tumor grades I II III	9(30.00)8(26.67)13(43.33)	−2.36 ± 0.84−0.77 ± 0.612.74 ± 0.77	0.02**
Tumor types Diffuse Intestinal	14(46.66)16(46.66)	1.68 ± 0.82−0.96 ± 0.82	0.06

* A higher ΔCt value indicates higher expression. ** Statistically significant.

**Table 5:** The relationship between cir_ITCH expression level (as divided into two groups based on the median of ΔCt) in GC tissues with clinicopathological parameters.

Name		Sequence (5’--> 3’)	Ta for PCR (°C)	Amplicon size
Divergentprimers	hcircITCH-F1	GTCCGGAACTATGAACAATG	55.9	207 bp	hcircITCH-R1	CTCTGTTGGCTCTTTGTCAC	hcircHIPK3-F1	TATGTTGGTGGATCCTGTTC	55.9	213 bp	hcirc HIPK3-R1	AACTGCTTGGCTCTACTTTG
	Convergent primers	hlinITCH-F1			GGTTCACCATCTGCCACTTC	60.7	169 bp	hlinITCH-R1			AGGGAGCTTGAGTTACAGGATT	hlin HIPK3-F1	GAAAGAAACTATCCACGGAC	54.4	177 bp	hlin HIPK3-R1	TATGACCTTTGTAGCACCTG

* A higher ΔCt value indicates higher expression. ** Statistically significant.

## 4. Discussion

In the current study, we explored the expression levels of *circHIPK3* and *cir_ITCH* in gastric cancer tissues compared to their paired adjacent noncancerous tissues as well as their clinicopathological significance.

Our results showed a significant underexpression of *circHIPK3* in GC tumoral tissues. Using RNA sequencing (RNA-seq), Zheng et al. (18) characterized an abundant circular RNA derived from the *HIPK3* gene, termed *circHIPK3*. They reported the significant overexpression of *circHIPK3* in liver cancer compared with their matched normal tissues. In 2017, Li et al. (24) measured the relative expression of *circHIPK3 *in 44 pairs of bladder cancer and normal bladder tissues. Consistent with their RNA-seq results, they observed a significant decrease of *circHIPK3 *levels in 79.5% of bladder tumoral tissues compared to their normal counterparts. Overexpression of *circHIPK3* in bladder cancer cell lines could suppress migration, invasion, and angiogenesis in vitro and inhibit bladder cancer growth and metastasis in vivo. Their findings support the tumor-suppressive activity of *circHIPK3* in bladder cancer. In agreement with the results of expression of this circular RNA in bladder cancer (24), we also observed the relative downregulation of *circHIPK3 *in gastric cancer tumoral tissues. The discrepancy between Li et al. (24) and our results vs. findings of Zheng et al. (18) on liver cancer may be ascribed to the potential tissue-specific expression and function of *circHIPK3.*

We furthermore analyzed the relative expression of *cir_ITCH *in GC and found that it was significantly underexpressed in tumoral tissues compared to the nontumoral adjacent tissues. The association between *cir_ITCH* and cancer was firstly described in a study on esophageal squamous cell carcinoma conducted by Li et al. (16). *cir_ITCH* was highly and significantly expressed in around 70.61% of tumoral tissues compared to their paired nontumoral tissues (16). Evaluation of *cir_ITCH* expression in colorectal cancer (17) showed its underexpression in 75.6% of cancerous tissues compared with the adjacent noncancerous tissues. In 2016, Wan et al. (15) evaluated the expression levels of *cir_ITCH* in a cohort of lung cancer compared to their paired adjacent normal tissues. Compared to non-tumoral lung tissues, *cir_ITCH* showed a significantly lower expression in 73.08% of tumoral tissues (15). Taken together, our result on gastric cancer is consistent with those of previous reports on ESCC (16), colorectal cancer (17), and lung cancer (15).

In 2017, Li et al. (24) reported a negative correlation of *circHIPK3* expression levels with bladder cancer grade, invasion, stage and the lymph nodes metastasis. In the same vein, we also observed that those patients with metastasis had a significant lower expression levels for this circular RNA. Gastric cancer patients with higher TNM stage or positive lymphatic invasion had lower levels of *circHIPK3,* although these correlations were not significant.

Furthermore, we found that the *cir_ITCH* expression level was significantly associated with age, metastasis, and cancer grades. Consistently, Wan et al. (15) reported a significant association between age and expression levels of this circular RNA in a cohort of lung cancer tissues.

## 5. Conclusions

In summary, our results showed that the expression of *cir_ITCH *and *circHIPK3* were significantly down regulated in GC tumoral tissues compared with their paired adjacent non-neoplastic counterparts. Further investigation of these preliminary results is needed to elucidate the precise functional roles of these two circular RNAs in gastric cancer pathogenesis. *cir_ITCH *and *circHIPK3* may serve in future as potential prognostic biomarkers in cancer.

## Acknowledgments

This original article was derived from the master’s thesis of Sara GHASEMI and was supported in part by a research grant number 395259 from Isfahan University of Medical Sciences, Isfahan, Iran. The funding body had no role in the design of the study and collection, analysis, and interpretation of the data and in writing the manuscript.
